# A Perspective on Chronic and Long-Lasting Anorexia Nervosa

**DOI:** 10.3389/fpsyt.2021.756669

**Published:** 2021-10-29

**Authors:** Maurizio Speciani, Yoram Barak, Hanafi Damanhuri, Diana De Ronchi, Fabio Panariello, Anna Rita Atti

**Affiliations:** ^1^Department of Biomedical and Neuromotor Sciences, Psychiatry, Bologna University, Bologna, Italy; ^2^Department of Psychological Medicine, Dunedin School of Medicine, University of Otago, Dunedin, New Zealand; ^3^Department of Biochemistry, Faculty of Medicine, UKM Medical Centre, Universiti Kebangsaan Malaysia, Kuala Lumpur, Malaysia

**Keywords:** anorexia nervosa, severe and enduring anorexia nervosa, older-age, long-lasting anorexia nervosa, role of emotions, affective and anxiety

## Abstract

Anorexia Nervosa (AN) is a severe eating disorder which typically develops in younger females. Many studies focus on this specific population, a majority of which will eventually partially or fully recover. A minority will become chronic despite extensive treatment. These patients are treatment-resistant and may not necessarily benefit from usual treatment. In this article we will reflect on possible mechanisms which may explain the maintenance of disease, and especially on the possible role of affective and anxiety disturbances. We will use, due to the lack of large-scale studies, data from risk and prognostic factors, treatment options and neurobiological correlates in chronic AN patients. Lastly, we will propose how these elements may advise further research and treatments.

## Introduction

Anorexia Nervosa (AN) is an eating disorder (ED) which significantly impacts younger females (1). It is characterized by low energy intake, fear of gaining weight, behavior which interferes with weight gain, and misperceptions regarding the patient's own body weight and shape (2) which significantly influence self-evaluation and misrepresent the severity of the illness to the patient. It typically develops in younger females, although cases emerging in the elderly population have been described (3). Two subtypes of AN are common: the binge-eating/purging subtype and the restricting subtype. Regardless of the subtype, patients reach extremely low weights. Affective and anxiety disturbances, such as depression, panic disorder and obsessive-compulsive disorder, are common comorbidities; substance abuse is also relatively frequent in these patients (2).

While a significant number of patients eventually recover, most reach partial of full remission only a number of years from the development of first symptoms (4). However, it has been estimated that more than 10% of AN patients (4) do not actually get better and eventually become chronic, as spontaneous recovery after more than 10 years of illness duration has been found to be rare, although it has been reported (5). There is, however, a paucity of studies on chronic AN, especially in the older population, possibly due to its relatively high remission rate after a few years of treatment (4). A definition of chronic AN has been developed, Severe and Enduring Anorexia Nervosa (SE-AN) (6), and some of its perpetuating factors have been suggested (7). While this definition is useful to describe peculiarities of chronic patients, due to the relatively broad criteria for SE-AN definition and the young age of emergence of symptoms, few studies actually focus on older-aged, long-standing AN.

We believe a different perspective on chronic, long-standing AN, based on evidence from neurobiological alterations, risk and prognostic factors, and available treatments, could suggest novel strategies to identify cases at higher-risk for chronicity. Patients with Anorexia Nervosa at a higher-risk of chronicity could share features which could be found in the elderly. This could, in turn, inform further studies and help the development of treatments aimed at both the younger and the older population.

In order to better understand chronic, long-standing AN, we will first describe two of its long-term definitions: Anorexia Tarda (AT), a form of Anorexia which occurs in the elderly, and SE-AN, focusing specifically on their limits.

Then, in order to better understand which factors may raise the risk of chronicity, we will look at the evidence regarding chronic cases of AN in order to integrate three areas of investigation: established risk and prognostic factors, which may intuitively influence the course of disease; evidence regarding treatments of chronic AN cases, as their effects (or lack thereof) may help better understand the neurobiological foundation of chronicity in AN; and lastly, evidence regarding neurobiological characteristics of chronic AN patients, in order to understand whether the integration of all three areas of investigation may suggest common features and mechanisms. This could help develop better treatment strategies which could benefit both the younger and the older population.

In conclusion, we will review the relevance and the limits of the available evidence, as the majority of the evidence focuses on the younger population, and we will suggest a different approach for future studies on the matter.

### Two Definitions of Chronic and Long-Lasting AN

Although associated with adolescence and young age, eating disorders may occur *de novo* and even recur in the elderly.

Already in the 1980s, awareness of “Anorexia in the Elderly” was raised as associated with psychiatric and physical comorbidities, or as a reduction in opioid feeding drive and excessive satiety that are a normal part of aging (8). The term “Anorexia Tarda” (AT) was then coined to differentiate Anorexia associated with depression, dementia, cancer, and other comorbidities from Anorexia as a distinct eating disorder in the elderly (9, 10). AT appears to be relatively rare among older women, with a population-based study reporting a 0.17% lifetime risk in women over 45 years old (11). It has not yet been established whether presentations of AT in older women represent a continuation of a lifelong illness or late onset of the disease: in the most comprehensive review to date (12), while AT was found to be more common than AN, comorbid depression was found in more than half of the older patients, which is in itself a risk factor for chronicity in AN (13). AT as a definition suffers from critical limits, as it lacks a consensus regarding age or duration of the illness, nor it differentiates *de novo* and recurrent cases.

Another definition recently gained traction: Severe and Enduring Anorexia Nervosa (SE-AN). Virtually unknown before 2010, it found its way in dozens of publications in the last decade. It is defined as AN which persists for more than 3–7 years (depending on the author) after appropriate treatments (6, 14).

Although this definition may apply to older patients who suffer from chronic anorexia nervosa, if AN emerges in adolescence it is possible that the SE-AN patient can be quite young, and thus data may not necessarily inform clinicians regarding treatment options for older-aged AN patients. As Herpertz-Dahlmann suggested in a recent commentary (7), definitive data is still lacking regarding AN and specifically SE-AN in order to identify specific risk factors which may have a role in the development of chronic AN over the course of the disease. As noted by Kaplan and Strober (15), “no predictive study of long-term risk of illness persistence” assessed in a comprehensive manner the role of the risk architecture linked to AN, such as traits, genetics and other phenotypes associated to the illness. Yet, one could argue that factors which have a role in the persistence of AN in patients with decades of illness may already be present in SE-AN patients, as the majority of AN patients go into partial or full remission in less than a decade from the emergence of the illness (4) and never become SE-AN patients. That would mean that data on SE-AN patients could still be useful to ascertain elements which might sustain AN from a pathophysiological point of view well into the old age. However, this definition has limitations as well, as no consensus has been reached on the duration of the illness (more than 3 or 7 years), and it does not differentiate age of onset, nor whether recurrences should be included.

Why do these patients become chronic, long-standing cases, while the majority of other AN patients eventually go into remission? This uncertainty may stem from a systemic issue. The widespread use of American Psychiatry Association and World Health Organization criteria for psychiatry research allows clinicians to compare patients in a cost-effective manner from all over the world by providing an invaluable common framework for diagnoses. However, a categorical approach primarily based on symptoms and life history may only partially take into account the neurobiological relationship between different diagnoses, traits and characteristics, especially when they present similarly.

Hence, which factors may have a role in fueling the chronicity of AN? In order to better define the characteristics of this population, and in the absence of more targeted studies, a look at established risk and prognostic factors may be useful. In the literature, a number of factors have been identified as negative predictors of illness, and they may be useful to understand chronic AN from a different perspective, as they may have identified common characteristics within the broader AN definition which correlate with a higher rate of chronicity.

### What Is the Evidence on Risk and Prognostic Factors in Anorexia Nervosa?

A number of risk and prognostic factors have been found to be involved in AN and its course. In Tozzi et al. (16) explored patients' perspectives on perceived causes of AN, and found that familial dysfunction (including emotional abuse), adverse experiences related to food (such as dieting, weight loss) and emotional duress (pressure, stress, frustration) were the most common perceived causes; a supportive relationship, personal growth (maturation) and therapy/counseling were the most frequently reported recovery factors. Bulik et al. (17) found that higher neuroticism raised the risk of developing AN; in Keski-Rahkonen et al. (13) found that pre-morbid depressive symptomatology predicted lower recovery rates in AN, and found that many unrecovered patients reported perfectionism and dissatisfaction with their partner.

Focusing on SE-AN patients, Le Grange et al. (18) compared Cognitive-Behavioral Therapy-AN (CBT-AN) and Specialist Supportive Clinical Management (SSCM) and found that lower age, shorter duration of illness, being employed, having better social adjustment and not taking any psychopharmacological medication were predictors of better outcome; eating disorder psychopathology, age, subtype of AN and depression were found as moderators of treatment. Regarding the role of comorbidities, extreme compulsiveness (4), obsessive-compulsive traits and OCD (19), depression (20, 21), and anxiety (22) also emerged as negative predictors for full recovery.

While these characteristics inform the clinician, the mechanism through which they relate to the course of the illness is still unclear. Do they correlate with a worse prognosis due to the added burden of a fully separate illness, do they reciprocally impair their healing processes, or maybe their coexistence actually defines a specific phenotype?

Furthermore, many of the aforementioned factors may be of limited use to the clinician who first approaches the patient. Factors such as family dysfunction, emotional abuse, the absence of a supportive relationship and previous, negative experiences related to food may already have affected the prognosis. Comorbidities, such as pre-morbid depressive symptoms or trait perfectionism, can be useful to stratify the risk of chronicity, but as pre-morbid factors they, too, may have already exerted their effect on the prognosis.

Although informative, as this categorical approach helps us clinicians to immediately recognize elements from life histories which may correlate with a higher risk of chronicity, we still do not know how they are related and the mechanism through which they influence the prognosis.

### What Is the Evidence on Treatment Options for Chronic, Long-Standing Anorexia Nervosa?

Can treatment options for chronic AN help us better understand the illness? The evidence in the available literature appears to be rather limited.

Despite being the disease with the highest fatality rate in psychiatry (23), there are still no approved pharmaceutical treatments for Anorexia Nervosa in Europe and in the United States of America. And for severe cases, effective treatment options are even less clear (24). However, a growing body of research investigated effective treatments for SE-AN.

Therapeutical interventions, in particular of a Cognitive-Behavioral imprint, such as CBT-AN (25), CBT-Enhanced, and Cognitive Remediation Therapy (26) appear to yield some results, although due to the uneven definition of SE-AN, mixed treatments and small sample sizes, their impact is still under investigation.

Evidence regarding pharmacological treatments is still quite limited. A trial of Dronabinol, a synthetic cannabinoid, showed evidence of weight gain in SE-AN patients but no change in AN symptoms (27), and a recent case report detailed full remission after 15 years of illness after a ketogenic diet and IV ketamine (5). A previous study on ketamine on treatment-resistant ED patients found longer remission of symptoms correlated to lower compulsion scores after ketamine infusions (28). Regarding brain stimulation therapies, studies on Deep Brain Stimulation found only small improvements in AN symptoms (29). Thus, data from treatment options for chronic cases is far from definitive, and does not appear to suggest a common pattern of efficacy among treatments, limiting our ability to formulate hypotheses regarding its mechanisms.

### What Is the Evidence on the Neurobiology of Anorexia Nervosa?

AN appears to have a significant biological basis, as its heritability has been estimated to be 0.56 (17), and a growing number of neurobiological alterations have been found to be related to AN.

Dopamine is a neurotransmitter which is notoriously involved in reward mechanisms (30). Reward pathways have been found to be altered in AN, for example when healthy controls perceived euphoria in a drug-induced spike of dopamine levels, AN patients (even when fully recovered) reported feelings of anxiety and unpleasantness, which may explain why AN patients report food-elicited sensations as aversive from a motivational standpoint (31). Furthermore, even recovered AN patient appear to have altered reward processing mechanisms, as they find more difficult to process information and feedback (32), and also to respond to motivational inputs (33), compared to controls. This difficulty in processing information, feedback and motivation, may alter their capacity to make a fully informed choice regarding their health, a critical issue especially in SE-AN patients (34). At the same time, AN patients appear to be able to outscore healthy controls in specific measures of cognition (35).

Serotoninergic systems appear to be altered as well. Polymorphisms of serotonin transporters have long been associated with a higher risk of developing AN (36). Serotonin is a neurotransmitter made from Tryptophan, which is an essential amino-acid taken from food, and possibly mediates many of the comorbidities that have been associated with AN such as depression (37), obsessive-compulsive disorder (38) and anxiety (39). Since starvation lowers the concentration of Tryptophan, it may be regarded as a control measure to limit neurotransmission (40), possibly due to an intolerance of symptoms such as thoughts, feelings and emotions of these illnesses. Other imbalances in neurotransmitters, hormones and peptides such as norepinephrine, estradiol, and ghrelin have been found in AN (31, 41).

Furthermore, imbalances in brain pathways may also mediate some of AN symptoms. It was found that the ability of AN patients to suppress hunger signals may be due to a reverse direction of signaling between hypothalamus and ventral striatum: in a recent study by Frank (42), signal in healthy controls was directed from the hypothalamus to the ventral striatum, while in AN patients signal direction was reversed.

## Discussion

### How to Integrate the Available Evidence?

While the available evidence still does not allow to define a phenotype of chronic, long-standing AN, due to the lack of rigorous large-scale studies on this population, we may attempt to integrate and interpret the available evidence in order to advise future studies and hypotheses on this population.

The question is: do cases of chronic, long-standing AN suffer from the same disease as patients who will eventually recover from AN, or do they suffer from a different psychopathological disorder which, while manifesting symptoms that meet the categorical criteria for AN, could be described as a distinct diagnostic entity in which eating symptoms mislead the diagnosis, thereby indirectly contributing to a higher resistance to treatment? And if so, what pathopsychological characteristics would this phenotype have?

In order to define this phenotype in the future, neurobiological alterations may be a helpful starting point, as research evidence is rapidly expanding. Nonetheless, evidence from treatment options and risk and prognostic factors may be useful as well, especially for chronic, older-aged AN patients which are not recovered. This is because some of the literature on AN neurobiology struggles with a limitation: to discern alterations that stem from long-term starvation, they compare patients with active illness and in remission (Recovered Anorexia Nervosa, or RECAN), thus in order to avoid the critical confounder of body starvation, alterations which persist after full recovery may actually had been already present before the illness developed, and could be deemed risk factors worth investigating. However, this approach does not necessarily portray the condition of chronic, still unrecovered cases.

In order to better understand which of the many alterations found from neurobiological studies could have a role in the development of chronic AN, risk and prognostic factors may be regarded as elements which fuel a dysregulation in a number of systems and correlate with chronicity. Although a nature vs. nurture argument could be made, considering the significant heritability of AN (17), it may be suggested that affective and anxiety systems could have a role in the persistence of AN, as among the aforementioned factors are found pre-morbid depressive symptoms, obsessional and obsessive-compulsive tendencies, and familial dysfunction, which is in itself a known risk factor for anxiety and depressive disorders (43, 44).

Although further studies are needed to better understand emotions, studies suggest the involvement of many structures in the brain, especially the amygdala, the prefrontal cortex (PFC), the orbitofrontal cortex (OFC), and the insula regarding motivation, interoception, emotions, anxiety and obsessive thoughts (45, 46). Thus, it may be suggested that alterations in the neurobiology of AN affect at least some of the structures which are involved in the process of generating emotions.

Studies on effective treatments are still lacking. However, therapy and especially Cognitive-Behavioral approaches seem to be somewhat effective in resistant cases, which may be due to their foundation of treatments which are particularly useful for affective and anxiety disorders. While it may be suggested that the improvement from Dronabinol treatment is mediated by cannabinoid effects on hunger signals (27), the positive effects found in the two small studies on ketamine (27, 28), a substance highly correlated to Esketamine, which was recently approved in Europe and in the United States for treatment-resistant depression, may further suggest a significant role of the depressive-anxiety spectrum on chronic, long-standing AN. Further studies on the significance of these mechanisms could explain why antidepressants are not effective treatments for AN (47).

Thus, at least part of the available evidence may suggest an involvement of affective and anxiety systems, the dysregulation of which may be worth investigating in chronic, long-standing AN patients.

### A Perspective on Chronic AN and Comorbidities, Which Came First: The Chicken or the Egg?

A critical missing element in order to define chronic AN patients is what is fueling the persistence of disease.

In our clinical experience, even general psychiatry services—and more so non-psychiatric medical services—are fast to refer to ED clinics as soon as disordered eating symptomatology is found, especially in younger patients. ED clinics then typically assess the patient and focus on the extent of ED symptoms to discern whether, from a categorical point of view, they can be classified as a true ED. As seen from the available literature on AN, specific ED treatment helps (36) and in the following months to years the majority of patients get better, and some may even reach full remission of ED symptoms, which is why patients are typically referred to treatment from specialists. As previously mentioned, however, a minority of patients becomes chronic.

The available evidence may suggest an involvement of affective and anxiety systems in the prognosis, which is sometimes found even before the development of symptoms. Nonetheless, the typical age of emergence of ED symptoms, adolescence and young adulthood, is hardly one where communication is clear between patients and their families. The weight loss, however, can be spotted from a distance, and patients are eventually brought to medical attention.

It may be possible that a portion of AN patients who will eventually become chronic first develop a disturbance and then use restriction and refusal of food as means of control of the effects of said disturbance. This could, with time, become a chronic, relatively stable equilibrium which is maintained by food restriction and may be worsened by eating, a process which may also be sustained by recently suggested novel mechanisms, such as imbalances in gut microbiota (48). As previously mentioned, neurobiological evidence suggests that AN patients react negatively to a number of pleasant stimuli, especially food-related stimuli (31). If this premise were to be confirmed by future studies, that would explain why treatments who mainly focus on eating symptomatology may not work as well on this specific subpopulation. As a consequence, therapies which encompass egosyntonicity and focus on measures such as motivation, have been proposed in the treatment of chronic AN (25).

## Conclusions

In conclusion, further studies are needed to better define the phenotype of the chronic, older-aged AN patient, as the SE-AN definition may include quite younger patients, and there is a substantial lack of evidence regarding AT. Thus, in order to better define the characteristics of long-lasting AN, a consensus on long-lasting AN and older-age AN, and especially their differences, must be reached. Moreover, the available literature may suggest the involvement of anxiety and affective disorders in the maintenance of AN, especially in long-lasting, chronic patients. However, due to the limited available evidence, further studies on the impact and prevalence of affective and anxiety disorders in AN patients are needed in order to clarify whether AN is (at least in part) masking and controlling symptoms of other disturbances which will eventually fuel the maintenance of disease, especially in chronic and treatment-resistant cases, possibly constituting a separate AN phenotype. It should also be noted that while our article attempts to describe different areas of investigations in chronic AN, it does not constitute a systematic review of the available evidence.

## Data Availability Statement

The original contributions presented in the study are included in the article/supplementary material, further inquiries can be directed to the corresponding author.

## Author Contributions

MS, YB, HD, DD, FP, and AA contributed to the conception and design of this manuscript. MS wrote the first draft of the manuscript. All authors contributed to manuscript revision, read, and approved the submitted version.

## Conflict of Interest

The authors declare that the research was conducted in the absence of any commercial or financial relationships that could be construed as a potential conflict of interest.

## Publisher's Note

All claims expressed in this article are solely those of the authors and do not necessarily represent those of their affiliated organizations, or those of the publisher, the editors and the reviewers. Any product that may be evaluated in this article, or claim that may be made by its manufacturer, is not guaranteed or endorsed by the publisher.

## References

[1] Nicholls D Viner R. Eating disorders and weight problems. BMJ. (2005) 330:950–3. 10.1136/bmj.330.7497.950 15845978 PMC556344

[2] American Psychiatric Association. Diagnostic and Statistical Manual of Mental Disorders: DSM-5, 5th Edn. Washington, DC: American Psychiatric Association (2013).

[3] Taylor IA Gill I Harripaul A. Unexplained weight loss in an 80-year-old woman. BMJ Case Rep. (2015) 2015:bcr2014206847. 10.1136/bcr-2014-206847 25618876 PMC4307086

[4] Strober M Freeman R Morrell W. The long-term course of severe anorexia nervosa in adolescents: survival analysis of recovery, relapse, and outcome predictors over 10-15 years in a prospective study. Int J Eat Disord. (1997) 22:339–60. 10.1002/(SICI)1098-108X(199712)22:4&lt;339::AID-EAT1&gt;3.0.CO;2-N 9356884

[5] Scolnick B Zupec-Kania B Calabrese L Aoki C Hildebrandt T. Remission from chronic anorexia nervosa with ketogenic diet and ketamine: case report. Front Psychiatry. (2020) 11:763. 10.3389/fpsyt.2020.00763 32848935 PMC7412264

[6] Hay P Touyz S. Classification challenges in the field of eating disorders: can severe and enduring anorexia nervosa be better defined? J Eat Disord. (2018) 6:41. 10.1186/s40337-018-0229-8 30555695 PMC6287358

[7] Herpertz-Dahlmann B. Serious and enduring anorexia nervosa from a developmental point of view: how to detect potential risks at an early stage and prevent chronic illness? Int J Eat Disord. (2020) 53:1313–4. 10.1002/eat.23329 32538483

[8] Morley JE Silver AJ. Anorexia in the elderly. Neurobiol Aging. (1988) 9:9–16. 10.1016/S0197-4580(88)80004-6 2898107

[9] Stein D Zemishlani C Shahal B Barak Y. Disordered eating in elderly female patients diagnosed with chronic schizophrenia. Isr J Psychiatry Relat Sci. (2005) 42:191–7. 16335632

[10] Landi F Picca A Calvani R Marzetti E. Anorexia of aging: assessment and management. Clin Geriatr Med. (2017) 33:315–23. 10.1016/j.cger.2017.02.004 28689565

[11] Larrañaga A Docet MF García-Mayor RV. High prevalence of eating disorders not otherwise specified in northwestern Spain: population-based study. Soc Psychiatry Psychiatr Epidemiol. (2012) 47:1669–73. 10.1007/s00127-012-0473-1 22237718

[12] Lapid MI Prom MC Burton MC McAlpine DE Sutor B Rummans TA. Eating disorders in the elderly. Int Psychogeriatr. (2010) 22:523–36. 10.1017/S1041610210000104 20170590

[13] Keski-Rahkonen A Raevuori A Bulik CM Hoek HW Rissanen A Kaprio J. Factors associated with recovery from anorexia nervosa: a population-based study. Int J Eat Disord. (2014) 47:117–23. 10.1002/eat.22168 24488835

[14] Broomfield C Stedal K Touyz S Rhodes P. Labeling and defining severe and enduring anorexia nervosa: a systematic review and critical analysis. Int J Eat Disord. (2017) 50:611–23. 10.1002/eat.22715 28444828

[15] Kaplan AS Strober M. Severe and enduring anorexia nervosa: can risk of persisting illness be identified, and prevented, in young patients? Int J Eat Disord. (2019) 52:478–80. 10.1002/eat.23019 30638275

[16] Tozzi F Sullivan PF Fear JL McKenzie J Bulik CM. Causes and recovery in anorexia nervosa: the patient's perspective. Int J Eat Disord. (2003) 33:143–54. 10.1002/eat.10120 12616580

[17] Bulik CM Sullivan PF Tozzi F Furberg H Lichtenstein P Pedersen NL. Prevalence, heritability, and prospective risk factors for anorexia nervosa. Arch Gen Psychiatry. (2006) 63:305–12. 10.1001/archpsyc.63.3.305 16520436

[18] Le Grange D Fitzsimmons-Craft EE Crosby RD Hay P Lacey H Bamford B. Predictors and moderators of outcome for severe and enduring anorexia nervosa. Behav Res Ther. (2014) 56:91–8. 10.1016/j.brat.2014.03.006 24727364

[19] Jagielska G Kacperska I. Outcome, comorbidity and prognosis in anorexia nervosa. Psychiatr Pol. (2017) 51:205–18. 10.12740/PP/64580 28581532

[20] Franko DL Tabri N Keshaviah A Murray HB Herzog DB Thomas JJ. Predictors of long-term recovery in anorexia nervosa and bulimia nervosa: data from a 22-year longitudinal study. J Psychiatr Res. (2018) 96:183–8. 10.1016/j.jpsychires.2017.10.008 29078155

[21] Eskild-Jensen M Støving RK Flindt CF Sjogren M. Comorbid depression as a negative predictor of weight gain during treatment of anorexia nervosa: a systematic scoping review. Eur Eat Disord Rev. (2020) 28:605–19. 10.1002/erv.2787 32886423

[22] Glasofer DR Muratore AF Attia E Wu P Wang Y Minkoff H. Predictors of illness course and health maintenance following inpatient treatment among patients with anorexia nervosa. J Eat Disord. (2020) 8:69. 10.1186/s40337-020-00348-7 33292619 PMC7709230

[23] Arcelus J Mitchell AJ Wales J Nielsen S. Mortality rates in patients with anorexia nervosa and other eating disorders. A meta-analysis of 36 studies. Arch Gen Psychiatry. (2011) 68:724–31. 10.1001/archgenpsychiatry.2011.74 21727255

[24] Wonderlich SA Bulik CM Schmidt U Steiger H Hoek HW. Severe and enduring anorexia nervosa: update and observations about the current clinical reality. Int J Eat Disord. (2020) 53:1303–12. 10.1002/eat.23283 32359125

[25] Hay PJ Touyz S Sud R. Treatment for severe and enduring anorexia nervosa: a review. Aust N Z J Psychiatry. (2012) 46:1136–44. 10.1177/0004867412450469 22696548

[26] Zhu J Yang Y Touyz S Park R Hay P. Psychological treatments for people with severe and enduring anorexia nervosa: a mini review. Front Psychiatry. (2020) 11:206. 10.3389/fpsyt.2020.00206 32265758 PMC7106475

[27] Andries A Frystyk J Flyvbjerg A Støving RK. Dronabinol in severe, enduring anorexia nervosa: a randomized controlled trial. Int J Eat Disord. (2014) 47:18–23. 10.1002/eat.22173 24105610

[28] Mills IH Park GR Manara AR Merriman RJ. Treatment of compulsive behaviour in eating disorders with intermittent ketamine infusions. QJM. (1998) 91:493–503. 10.1093/qjmed/91.7.493 9797933

[29] Kotilahti E West M Isomaa R Karhunen L Rocks T Ruusunen A. Treatment interventions for severe and enduring eating disorders: systematic review. Int J Eat Disord. (2020) 53:1280–302. 10.1002/eat.23322 32488936

[30] Arias-Carrión O Stamelou M Murillo-Rodríguez E Menéndez-González M Pöppel E. Dopaminergic reward system: a short integrative review. Int Arch Med. (2010) 3:24. 10.1186/1755-7682-3-24 20925949 PMC2958859

[31] Higgins A. The neurobiology of anorexia nervosa. Anorexia and Bulimia Nervosa. (2018) 1:3–4. 10.5772/intechopen.82751

[32] Wagner A Aizenstein H Venkatraman VK Fudge J May JC Mazurkewicz L. Altered reward processing in women recovered from anorexia nervosa. Am J Psychiatry. (2007) 164:1842–9. 10.1176/appi.ajp.2007.07040575 18056239

[33] Friederich HC Kumari V Uher R Riga M Schmidt U Campbell IC. Differential motivational responses to food and pleasurable cues in anorexia and bulimia nervosa: a startle reflex paradigm. Psychol Med. (2006) 36:1327–35. 10.1017/S0033291706008129 16790080

[34] van Elburg A Danner UN Sternheim LC Lammers M Elzakkers I. Mental capacity, decision-making and emotion dysregulation in severe enduring anorexia nervosa. Front Psychiatry. (2021) 12:545317. 10.3389/fpsyt.2021.545317 33776810 PMC7991306

[35] Seidel M Brooker H Lauenborg K Wesnes K Sjögren M. Cognitive function in adults with enduring anorexia nervosa. Nutrients. (2021) 13:859. 10.3390/nu13030859 33808018 PMC7998517

[36] Sadock BJ Sadock VA Ruiz P. Kaplan & Sadock's Comprehensive Textbook of Psychiatry: 50th Anniversary Edition, 10th Edn. Philadelphia, PA: Lippincott Williams & Wilkins (2017).

[37] Grahame-Smith DG. Serotonin in affective disorders. Int Clin Psychopharmacol. (1992) 6(Suppl. 4):5–13. 10.1097/00004850-199206004-00004 1431011

[38] Barr LC Goodman WK Price LH McDougle CJ Charney DS. The serotonin hypothesis of obsessive compulsive disorder: implications of pharmacologic challenge studies. J Clin Psychiatry. (1992) 53(Suppl.):17–28. 1532961

[39] Charney DS Deutch A. A functional neuroanatomy of anxiety and fear: implications for the pathophysiology and treatment of anxiety disorders. Crit Rev Neurobiol. (1996) 10:419–46. 10.1615/CritRevNeurobiol.v10.i3-4.70 8978989

[40] Kaye WH Barbarich NC Putnam K Gendall KA Fernstrom J Fernstrom M. Anxiolytic effects of acute tryptophan depletion in anorexia nervosa. Int J Eat Disord. (2003) 33:257–67; discussion 268–270. 10.1002/eat.10135 12655621

[41] Rossi E Cassioli E Gironi V Idrizaj E Garella R Squecco R. Ghrelin as a possible biomarker and maintaining factor in patients with eating disorders reporting childhood traumatic experiences. Eur Eat Disord Rev. (2021) 29:588–99. 10.1002/erv.2831 33939220 PMC8251850

[42] Frank GKW DeGuzman MC Shott ME Laudenslager ML Rossi B Pryor T. Association of brain reward learning response with harm avoidance, weight gain, and hypothalamic effective connectivity in adolescent anorexia nervosa. JAMA Psychiatry. (2018) 75:1071–80. 10.1001/jamapsychiatry.2018.2151 30027213 PMC6233809

[43] Lin X Zhang Y Chi P Ding W Heath MA Fang X. The mutual effect of marital quality and parenting stress on child and parent depressive symptoms in families of children with oppositional defiant disorder. Front Psychol. (2017) 8:1810. 10.3389/fpsyg.2017.01810 29104548 PMC5654759

[44] Spinazzola J Hodgdon H Liang LJ Ford JD Layne CM Pynoos R. Unseen wounds: the contribution of psychological maltreatment to child and adolescent mental health and risk outcomes. Psychol. Trauma Theory Res. Pract. Policy. (2014) 6:S18–28. 10.1037/a0037766

[45] Celeghin A Diano M Bagnis A Viola M Tamietto M. Basic emotions in human neuroscience: neuroimaging and beyond. Front Psychol. (2017) 8:1432. 10.3389/fpsyg.2017.01432 28883803 PMC5573709

[46] Grant JE Chamberlain SR. Exploring the neurobiology of OCD: clinical implications. Psychiatr Times. (2020). 32624642 PMC7334048

[47] Marvanova M Gramith K. Role of antidepressants in the treatment of adults with anorexia nervosa. Ment Health Clin. (2018) 8:127–37. 10.9740/mhc.2018.05.127 29955558 PMC6007635

[48] Igudesman D Sweeney M Carroll IM Mayer-Davis EJ Bulik CM. Gut-brain interactions: implications for a role of the gut microbiota in the treatment and prognosis of anorexia nervosa and comparison to type I diabetes. Gastroenterol Clin North Am. (2019) 48:343–56. 10.1016/j.gtc.2019.04.003 31383275 PMC6686879

